# Excessive Intake of Longan Arillus Alters gut Homeostasis and Aggravates Colitis in Mice

**DOI:** 10.3389/fphar.2021.640417

**Published:** 2021-03-26

**Authors:** Huimin Huang, Mingxing Li, Yi Wang, Xiaoxiao Wu, Jing Shen, Zhangang Xiao, Yueshui Zhao, Fukuan Du, Yu Chen, Zhigui Wu, Huijiao Ji, Chunyuan Zhang, Jing Li, Qinglian Wen, Parham Jabbarzadeh Kaboli, Chi Hin Cho, Shengpeng Wang, Yitao Wang, Yisheng He, Xu Wu

**Affiliations:** ^1^ Laboratory of Molecular Pharmacology, Department of Pharmacology, School of Pharmacy, Southwest Medical University, Luzhou, China; ^2^ South Sichuan Institute of Translational Medicine, Luzhou, China; ^3^ Department of Pharmacy, The Affiliated Hospital of Southwest Medical University, Luzhou, China; ^4^ Faculty of Medicine, School of Biomedical Sciences, The Chinese University of Hong Kong, Hong Kong, China; ^5^ Department of Oncology and Hematology, Hospital (T.C.M) Affiliated to Southwest Medical University, Luzhou, China; ^6^ Department of Oncology, The Affiliated Hospital of Southwest Medical University, Southwest Medical University, Luzhou, China; ^7^ State Key Laboratory of Quality Research in Chinese Medicine, Institute of Chinese Medical Sciences, University of Macau, Macao, China

**Keywords:** longan, free sugar, inflammatory bowel disease, gut microbiota, short-chain fatty acid

## Abstract

**Background:** Longan is the fruit of *Dimocarpus longan* Lour. and the longan arillus has long been used in traditional Chinese medicine possessing various health benefits. However, the excessive intake of longan is found in daily life to cause “*shanghuo*” syndrome. “*Shanghuo*” has been linked to increased disease susceptibility. The present study thus aimed to investigate the toxicological outcomes after excessive longan treatment.

**Methods:** Longan extract at a normal dosage of 4 g/kg and two excess dosages of 8 and 16 g/kg was orally administered to normal C57BL/6J mice for two weeks or to C57BL/6J mice with DSS-induced colitis. Mouse gut microbiome were analyzed by 16S rRNA sequencing. Short chain fatty acid (SCFA) contents in colonic contents were measured by GC-MS. Colon tissue was used for histopathological observation after H and E staining, detection of protein expression by western blot, analysis of gene expression by qPCR, and detection of apoptotic cells by TUNEL assay. ELISA was used for biochemical analysis in serum.

**Results:** In normal mice, repeated longan intake at excess doses, but not the normal dose, increased infiltration of inflammatory cells, elevated serum levels of TNF-α and IL-6 and reduced production of SCFAs. In DSS-induced colitic mice, longan intake at 4 g/kg did not promote colitis in mice, while excessive longan (8 or 16 g/kg) aggravated colitis in mice, showing increased inflammation, more serious histological abnormalities, increased gut permeability, and increased epithelia injury when compared to DSS alone. Excessive longan induced a significant reduction of microbial diversity in colitic mice, accompanied with aggravated alterations of DSS-associated bacteria including the increase of Proteobacteria phylum and genera of *Bacteroides*, *Akkermansia*, *Turicibacter* and *Escherchia-Shigella*, and the decrease of *norank_f__Muribaculaceae*. The changed microbial compositions were accompanied with decreased SCFAs when longan was supplemented with DSS. The aggravated colon injury by excessive intake of longan in colitic mice was tightly correlated with the altered microbial communities and decreased SCFAs production.

**Conclusion:** Excessive longan intake disturbs gut homeostasis and aggravates colitis via promoting inflammation and altering gut microbe compositions and associated metabolism in mice. Our findings warrant rational longan arillus consumption as a dietary supplement or herbal medicine.

## Background

Longan is the fruit derived from *Dimocarpus longan* Lour. (*Sapindaceae* family), which is mostly distributed in Asia area, such as China, Vietnam, Thailand, and India. The dried longan pulp (longan arillus; also called *long-yan-rou* in Chinese) has long been used as a tonic in traditional Chinese medicine (TCM) for improving palpitations, forgetfulness, and insomnia (Zhang et al., 2020). Previous studies have shown that longan arillus possessed antioxidant, anti-inflammatory, immunoenhancing, anti-fatigue and anti-cancer activities (Chen et al., 2010; Park et al., 2010; Zhang et al., 2020). In clinical reports, longan arillus is well-tolerated at normal doses in human, with very few cases of allergy reported by orally taking longan fruit (Cheng and Huo, 2009). One report demonstrated that the sugar extract (Centrifugation followed by calcium hydroxide treatment and condensation) of fresh longan pulp had no acute (at 20 g/kg) and chronic toxicity (at 2.5 g/kg) in rats (Chiranthanut et al., 2020).

However, the excessive intake of longan fruits or dried longan has been found in daily life to cause “*shanghuo*”, a status described by TCM theory with typical symptoms of oral dryness, oral ulcers, gum bleeding and swelling. “*Shanghuo*” is actually a concept that describes an abnormal internal status of body, manifested by disruption of microenvironment homeostasis and induction of inflammation (Rongrong and Hiroshi, 2008; Pan et al., 2020). Notably, “*shanghuo*” status has been highlighted to increase disease susceptibility (Pan et al., 2020).

Chemically, longan arillus contains bioactive constituents of polysaccharides (17–24%, w/w) (Li, 2012), flavonoids (total flavonoids, 0.027%) (Zhang et al., 2018), vitamins and others. Apart from the non-caloric bioactive components, high level of free sugars including fructose (11.9–24.6%), glucose (5.6–22.8%) and sucrose (21.4–56.1%) are found (Zhong et al., 2011). Notably, dietary free sugar have been suggested as one of the most important risk factors for overweight, dental caries and non-communicable diseases (Te Morenga et al., 2013; Bray and Popkin, 2014). Previous reports also highlighted that dietary free sugars damaged gut microbiome and promoted colitis in mice (Khan et al., 2020). Free sugars, fructose in particular, were demonstrated to disrupt the gut-liver axis, possibly through increased gut permeability and altered gut microbiota (Todoric et al., 2020; Zhao et al., 2020). Although it is widely accepted that intake of fruit has health benefit in human and is correlated with decreased risk of cardiovascular disease and some cancers, recently, a large-scale population-based prospective cohort study showed that the consumption of sugary drinks, even pure fruit juice, was positively associated with the increased risk of overall cancer (Chazelas et al., 2019). There is rare evidence for the association of intake of high free sugar-containing fruit and risk of diseases (particularly within gut-liver axis) such as colitis. It is thus of primary interest to investigate whether excessive longan intake may result in aggravation of certain diseases.

Therefore, in the present study, we evaluated the toxicological outcomes after normal or excessive longan supplementation in normal mice and mice with dextran sulfate sodium (DSS)-induced colitis. The results would add knowledge into the understanding of longan-related “*shanghuo*”, provide scientific basis for colitis associated with excessive longan consumption and warrant rational longan intake among general public as either a dietary supplement or a tonic.

## Materials and Methods

### Chemicals and Reagents

Dextran sulfate sodium (DSS; MW. 36–50 kDa) was purchased from International Laboratory (United States). Distilled water was prepared from Milli-Q system (Millipore).

### Preparation of Longan Extract

The extraction of dried longan arillus (1 kg; purchased from Kangmei Pharmaceutical Co., Ltd., Guangdong, China) was conducted using boiling water for three times (1 h each time) followed by lyophilization. The longan arillus extract (LE) was stored at -80°C until further analysis. Determination of free sugars by HPLC showed that the contents were fructose 17.6%, glucose 13.7% and sucrose 37.2% (*w/w*) (Supplementary Figure S1).

### Animals

Specific-pathogen-free male C57BL/6J mice (4 weeks-old; Beijing HFK Bio-Technology Co., Ltd.) were housed in ventilated cages (five animals per cage) at the animal center of Southwest Medical University under controlled conditions (22 ± 2°C; 55–60% humidity; and 12/12 h light/dark cycle) with free access to sterilized standard chow and tap water. The care of animals and all experimental procedures were conducted according to the NIH guidelines and were approved by the Committee on Use and Care of Animals of Southwest Medical University (Reference No., 2020226). All mice were adapted to the environment for at least 1 week before the experiment.

### Normal Mouse Experiment

Mice fed with normal diet (#LAD0011; Trophic Animal Feed High-Tech Co., Ltd., Jiangsu, China) were randomly allocated into four groups (*n* = 7 in control group; *n* = 5 in each of LE-L, LE-M and LE-H groups). Mice in LE-L, LE-M, and LE-H groups were orally administered with LE (dissolved in sterilized distilled water) at low, medium and high dosage of 4, 8, and 16 g/kg, respectively, every other day for 2 weeks. Mice in control group (Ctrl) received orally distilled water.

At the day before the end of animal experiment, mouse fecal samples were collected at 15:00–17:00 to minimize possible circadian effects. Samples were immediately placed in sterilized tubes on ice and transferred to −80°C storage within 2 h. Mice were anaesthetized with ether and blood were collected through cardiac puncture. Mice were then sacrificed by cervical dislocation immediately after the blood collection, followed by collection of colonic contents, colon and liver tissue samples. Blood samples were further centrifuged after coagulation at 4°C at sequential 3,000 and 12,000 rpm/min for 5 and 10 min to obtain serum samples, which were stored at −80°C. Fresh colon and liver tissues were washed with ice-cold PBS and stored at −80°C.

### DSS-Induced Colitic Mice

Mice fed with normal diet (#LAD0011, Trophic Animal Feed High-Tech Co., Ltd., Jiangsu, China) were randomly allocated into different groups, namely Ctrl, DSS, DSS + LE-L, DSS + LE-M, and DSS + LE-H groups (*n* = 10 per group). To induce acute colitis, mice were fed with 3.5% (w/v) DSS supplemented in distilled drinking water for five consecutive days (Day first-fifth). Colitic mice were orally gavaged with LE every other day at 0, 4, 8, and 16 g/kg, respectively, in DSS, DSS + LE-L, DSS + LE-M, and DSS + LE-H groups from day 1st to day 14th. Ctrl mice were given normal drinking water, and were orally gavaged with distilled water every other day from day 1st to day 14th.

Body weight of each mouse was weighed every other day. DSS-treated mice experienced rapid body weight loss with some of mice died between day 9th and 12th. To ensure animal benefit, we stopped the experiment at day 12th. At day 12th, fecal specimen of each mice was collected at 15:00–17:00 and stored at −80 °C. At day 13th, mice were anaesthetized with ether and blood were collected through cardiac puncture. Mice were then sacrificed by cervical dislocation immediately after the blood collection. The length of colon (including cecum to rectum) was measured. Mouse serum, colonic contents, colon and liver samples were collected and stored at −80°C.

### Biochemical Analysis

Serum levels of TNF-α and IL-6 were determined by ELISA kits (Elabscience Biotechnology Co., Ltd.) according to the manufacturer’s instruction. Lipopolysaccharide (LPS) level in serum was detected by kit obtained from CUSABIO Technology LLC.

### H and E Staining

Formalin-fixed paraffin-embedded sections were stained with H and E stain as previously reported (Zhu et al., 2017). H and E sections were inspected using Nikon Eclipse Ts2R + FL microscope.

For colitis mice, histopathological scores (0–9, from the least to most severe damage) were examined based on the scoring rule: inflammatory cell infiltration (0–3), crypt distortion (0–3) and colon mucous membrane detachment (0–3).

### TUNEL Staining

Paraffin sections were dewaxed using xylene, and were permeabilized with 20 μg/mL proteinase K solution (#ST533, Beyotime) for 25 min. Terminal deoxynucleotidyl transferase (TdT) and dUTP (#C1088, Beyotime) were then added and incubated in a humidified chamber at 37°C for 1 h followed by nuclei staining with DAPI (#H-1200, Vector Laboratories Inc.). Sections were inspected by a Nikon Eclipse Ts2R-FL fluorescence microscope. Image J software (Version 1.48v, NIH, United States) was used to calculate the number of TUNEL positive cells.

### Quantitative PCR

RNA was extracted from liver and colon samples using TRIzol reagent (Life technologies). Reverse transcription of RNA (1 μg) into cDNA was conducted using PrimeScript RT reagent kit (TaKaRa) according to the manufacturer’s protocol. Reverse transcription PCR was performed using PrimeScript RT reagent kit (TaKaRa). Quantitative PCR (qPCR) analysis was carried out in an CFX ConnectTM Real Time system (Bio-Rad) using SYBR Green Real Time PCR kit (applied Biosystems, life technologies).

Primer sequences for mouse *GAPDH*, *TNF-α* and *IL-1β* for qPCR reactions were as follows: *GAPDH*, 5′-AGG​AGC​GAG​ACC​CCA​CTA​ACA-3′ (forward), 5′-AGG​GGG​GCT​AAG​CAG​TTG​GT-3′ (reverse); *TNF-α*, 5′-AGC​CGA​TGG​GTT​GTA​CCT​TG-3′ (forward), 5′-ATA​GCA​AAT​CGG​CTG​ACG​GT-3′ (reverse); *IL-1β*, 5′-CCG​TGG​ACC​TTC​CAG​GAT​GA-3′ (forward), 5′-GGG​AAC​GTC​ACA​CAC​CAG​CA-3′ (reverse).The relative level of target gene was quantitated using ΔΔCt method, expressing as 2^−ΔΔCt^.

### Western Blot

Protein samples (28 µg) extracted from colon samples were electrophoresed on 10% SDS-PAGE gels (#PG112, EpiZyme) and then transferred onto polyvinylidene fluoride (PVDF) membranes. After incubation with anti-ZO-1 monoclonal antibody (1:1,000; #ab96587, Cell Signaling Technology Inc.), anti-β-actin antibody (1:3,000; #AF0003, Beyotime) at 4°C overnight, blots were then incubated with horseradish peroxidase conjugated anti-mouse (1:3,000, #A0208, Beyotime) or anti-rabbit antibodies (1:3,000; #A0216, Beyotime) at room temperature for 2 h. Protein bands were immunodetected using enhanced chemiluminescence reagent (#170–5,061, Bio-Rad). The expression level of ZO-1 was obtained by gray value analysis using Image J software.

### Microbial DNA Extraction and PCR Amplification

Microbial DNA extraction, PCR amplification, and purification and quantification of PCR products were conducted as we previously reported (Yin et al., 2020).

### Illumina MiSeq Sequencing

Purified amplicons were pooled in equimolar and paired-end sequenced (2 × 300) on an Illumina MiSeq platform (Illumina) based on the standard protocols by Majorbio Bio-Pharm Technology Co. Ltd. (Shanghai, China).

### Processing of Sequencing Data

Analysis of the fecal microbial community was performed using the free online platform of Majorbio Cloud Platform (www.majorbio.com) and Microbiomeanalyst (https://www.microbiomeanalyst.ca/). Raw fastq files were demultiplexed, quality-filtered by Trimmomatic and merged by FLASH with the criteria as we previously described (Yin et al., 2020).

Operational taxonomic units (OTUs) were clustered with 97% similarity cut off using UPARSE (version7.1) and chimeric sequences were identified and removed using UCHIME. The taxonomy of each 16S rRNA gene sequence was analyzed by RDP Classifier algorithm against the Silva (SSU123) 16S rRNA database using confidence threshold of 70%.

Rarefaction curves and α diversity were analyzed using mothur v1.30.1 and β diversity was determined using QIIME. Partial least squares discriminant analysis (PLS-DA) was performed in R tools using package mixOmics. Data structure was analyzed by principal co-ordinates analysis (PCoA) using the Bray-Curtis dissimilarity matrices. Linear discriminant analysis (LDA) coupled with effect size (LEfSe) was achieved using LEfSe program in MicrobiomeAnalyst (https://www.microbiomeanalyst.ca/).

Based on 16S rRNA sequencing data, Tax4Fun, an open-source R package, was used to predict functional changes of microbial communities mapping with Kyoto Encyclopedia of Genes and Genomes (KEGG) reference database.

### Short-Chain Fatty Acids Determination

The determination of free fatty acids (Acetic acid, propionic acid, isobutyric acid, butyric acid, isovaleric acid, valeric acid and caproic acid) was performed using the Thermo TRACE 1310-ISQ LT gas chromatography coupled with mass spectrometry (GC-MS). An Agilent HP-INNOWax column (30 m × 0.25 mm, ID 0.25 μm) (Agilent Technologies, United States) was used for chromatographic separation. Helium was the carrier gas operated at 1 ml/min. Injection was performed in split mode at 10: 1 with an injection volume of 1 μL, with an injector temperature of 250°C. The temperature of the ion source, interface, and quadrupole were set at 230, 250, and 150°C, respectively. The gradient program for column temperature was as follows: increasing from 90 to 120°C at 10°C/min, to 150°C at 5°C/min, and finally to 250°C at 25°C/min and kept for 2 min (total 15 min). The detector was operated in electron impact ionization mode (electron energy 70 eV) using selected ion monitoring (SIM) mode. Isocaproic acid was used as an internal standard.

For sample preparation, an aliquoted of 50 mg colonic content was vortex mixed with 15% phosphoric acid (50 μL), 125 μg/ml internal standard (100 μL), and ether (400 μL) for 1 min, followed by centrifugation at 12,000 rpm for 10 min at 4°C. The supernatant was used for analysis.

Quantification of acetic acid, propionic acid, isobutyric acid, butyric acid, isovaleric acid, valeric acid and caproic acid were validated with linearity, limit of quantification, intra-day and inter-day precision, repeatability, and recovery. The results are displayed in Supplementary Tables S1, S2, which showed the reliability and accuracy of detection method.

### Statistical Analysis

Statistical difference was assessed by GraphPad Prism software based on unpaired student’s *t* test (for comparison between two groups) or one-way ANOVA with a post hoc Tukey test (for comparison among three or more groups). All the results are statistically significant at a *p* value less than 0.05.

## Results

### Excessive Intake of Longan Induces Inflammation in Mice

We firstly investigated the impact of longan intake at varied doses on mice. The doses of longan extract (LE) used for mice were set as 4, 8, and 16 g/kg (approximately equivalent to human doses of 20–28, 40–56, and 80–112 g dried Longan arillus, respectively). The 4 g/kg LE in mice (LE-L group) was generally at the maximum recommended dose, while the dosages at 8 g/kg (LE-M group) and 16 g/kg (LE-H group) were considered as excessive LE intake.

**FIGURE 1:**
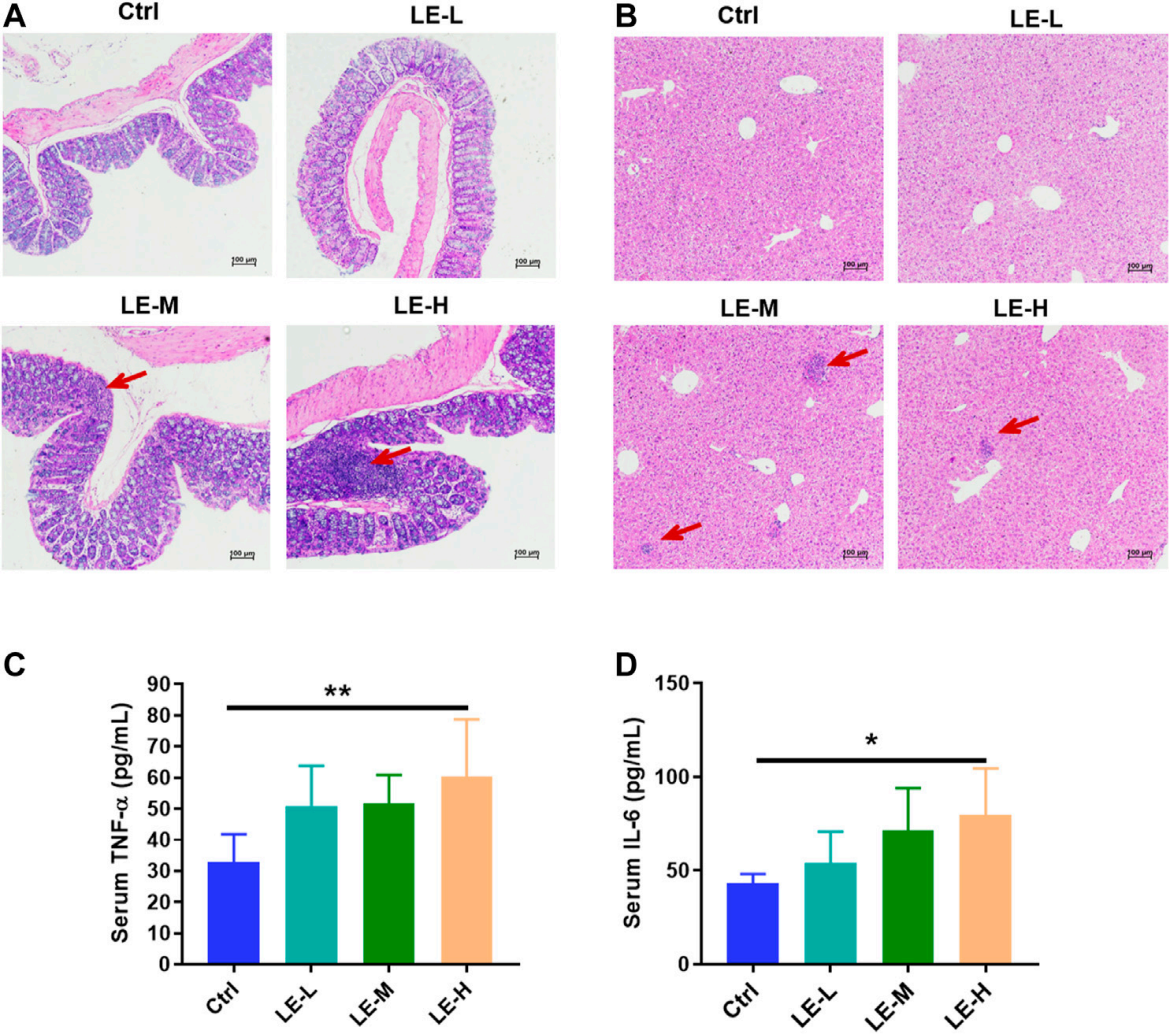
Excessive longan intake promotes inflammation in mice. H and E staining of colon sections **(A)** and liver sections **(B)** after 2 weeks of longan extract (LE) treatment. The oral dosage of LE for Ctrl, LE-L, LE-M, and LE-H group are 0, 4, 8 and 16 g/kg, respectively. Red arrow shows inflammatory cell infiltration. Serum levels of TNF-α **(C)** and IL-6 **(D)** in mice after 2 weeks of LE intake. Data are presented as mean ± SD (*n* = 7 for Ctrl group; *n* = 5 for other groups). *p < 0.05, **p < 0.01, compared to Ctrl.

After a 2 weeks oral administration of LE, mice in LE-M group (Three out of six mice) and LE-H group (Three out of six mice) demonstrated increased infiltration of inflammatory cells in colon (Figure 1A) and liver (Figure 1B) samples. Besides, compared to Ctrl mice, levels of the proinflammatory factors of TNF-α (Figure 1C) and IL-6 (Figure 1D) in serum were significantly elevated in the LE-H group (*p* < 0.05) with a 2 weeks LE treatment. By comparison, mice in LE-L group had no sign of inflammatory cell infiltration in colon and liver samples after 2 weeks of low-dose LE (4 g/kg) treatment, which was verified by unchanged serum levels of TNF-α and IL-6 (Figures 1A–D). The results indicated that excess LE (8 or 16 g/kg) for 2 weeks could induce an inflammatory status in mouse colons and livers. The results indicated that repeated LE treatment (particularly for the excess doses in LE-M and LE-H groups) induced a proinflammatory status in mice.

### Excessive Intake of Longan Mediates Rearrangement of Intestinal Microbial Structure in Mice

**FIGURE 2:**
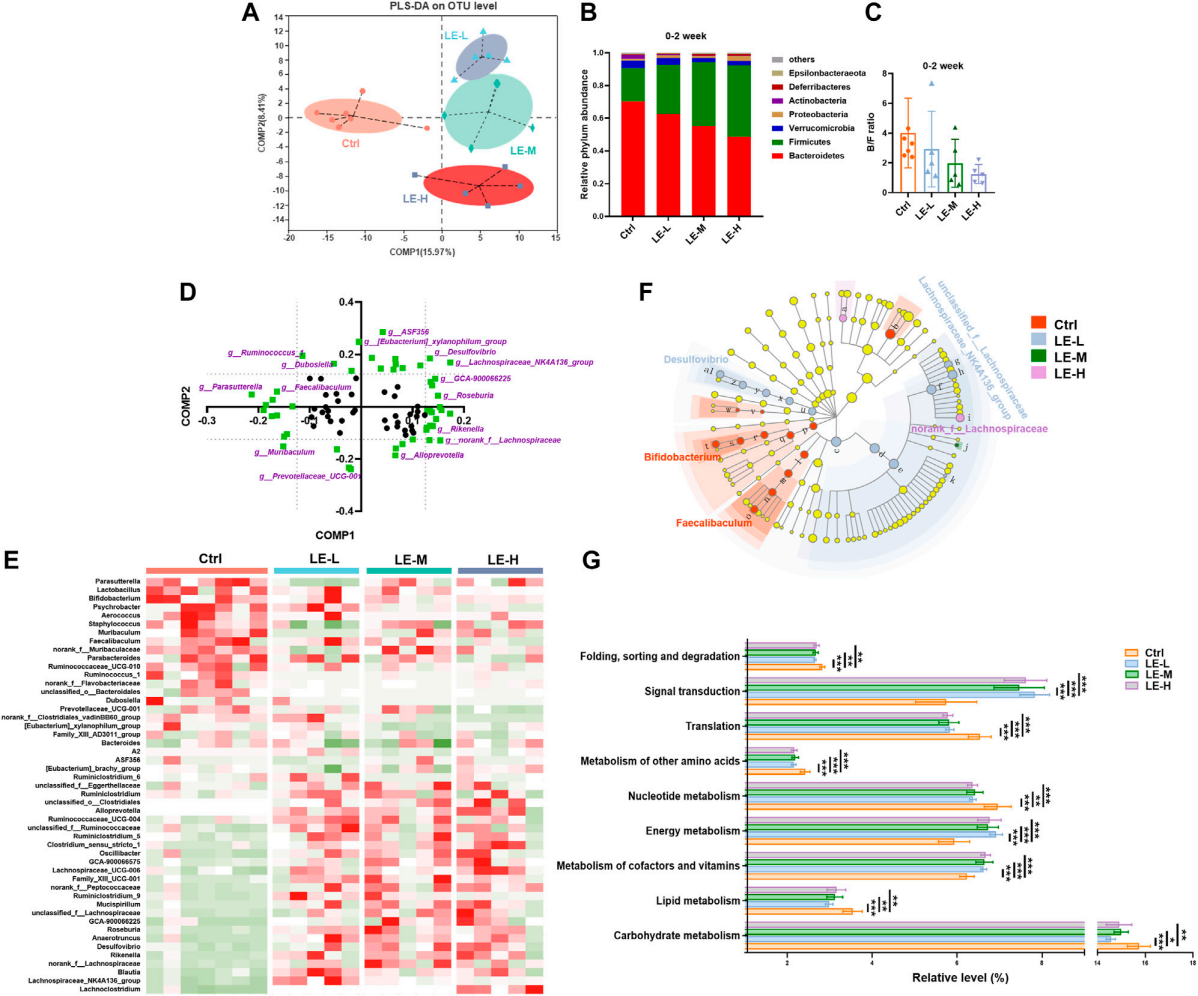
Excessive longan intake induces structural changes of intestinal microbiota in mice. **(A)** PLS-DA plot based on OTU level after two weeks of LE administration **(B)** Phylum level differences among groups. **(C)** Ratio of Bacteroidetes/Firmicutes (B/F). **(D)** Loading plot of bacterial genus contributing to PLS-DA grouping of samples. To distinguish the most significant contributors (indicated by purple), a threshold (>0.125 or <0.125) was set for component 1 (Comp 1) and component 2 (Comp 2). **(E)** Heatmap analysis based on identified significant contributors. **(F)** Cladogram for Linear discriminant analysis (LDA) score and LDA effect size (LEfSe) analysis. **(G)** Functional features of the resulting bacterial communities with LE intake predicted by Tax4Fun. Data are presented as mean ± SD (*n* = 7 for Ctrl group; *n* = 5 for other groups). *p < 0.05, **p < 0.01, ***p < 0.001, compared to Ctrl.

We then examined the mice on colonic microbiota. As shown in PLS-DA plot (based on OTU level), in 2 weeks LE treatment (Figure 2A), samples from Ctrl, LE-L, LE-M, and LE-H groups were clearly separated, indicating that LE markedly mediated structural changes of gut microbiota. At phylum level, a gradual increase in abundance of Firmicutes and a decrease in Bacteroidetes was correlated with the increasing LE intake (Figure 2B). As downregulated Bacteroidetes/Firmicutes (B/F) ratio has been suggested as an indicator of several pathological conditions (Turnbaugh et al., 2009), here we found that the B/F ratio was decreased along with the increased LE dosage (Figure 2C), but with no statistical difference. At genus level, with increasing dosages, *norank_f__Lachnospiraceae*, *unclassified_f__Lachnospiraceae*, *Lachnospiraceae_NK4A136_group*, *Desulfovibrio*, *Ruminiclostridium_9*, *Lachnoclostridium*, *Rikenella*, *Anaerotruncus* were increased, while *Bifidobacterium*, *Parasutterella*, and *Parabacteroides* were decreased (Supplementary Figure S2).

In order to distinguish the predominant taxon, we further performed heatmap and LEfSe analysis. The loading plot for PLS-DA analysis showed the significance of bacterial genera contributing to discriminating different groups (Figure 2D). Heat map of the most significant ones (highlighted with purple color, with Comp1 or Comp2 values >0.125, or <−0.125) demonstrated that there were remarkably different patterns of generic abundance across different groups (Figure 2E). LEfSe was used to produce a cladogram to show the specific bacteria associated with LE treatment. While the Ctrl group showed enriched *f__Muribaculaceae*, *Bifidobacterium*, and *Faecalibaculum*, the 2 weeks LE treatment altered microbiota composition manifested by enriched *Desulfovibrio*, *unclassified_f__Lachnospiraceae*, *Lachnospiraceae_NK4A136_group*, *norank_f__Lachnospiraceae*, and *f__Prevotellaceae* in all LE treatment groups (LDA score >4) (Figure 2F).

Furthermore, functional analysis by Tax4Fun revealed that the 2 weeks of LE treatment significantly enriched the annotated KEGG pathways related to signal transduction and energy metabolism (Figure 2G). On the other hand, several metabolic pathways regarding the carbohydrate metabolism, lipid metabolism and metabolism of other amino acids were significantly decreased in LE treated group (Figure 2G). It is suggested that LE-fed mice specifically showed altered metabolic pathways.

**FIGURE 3:**
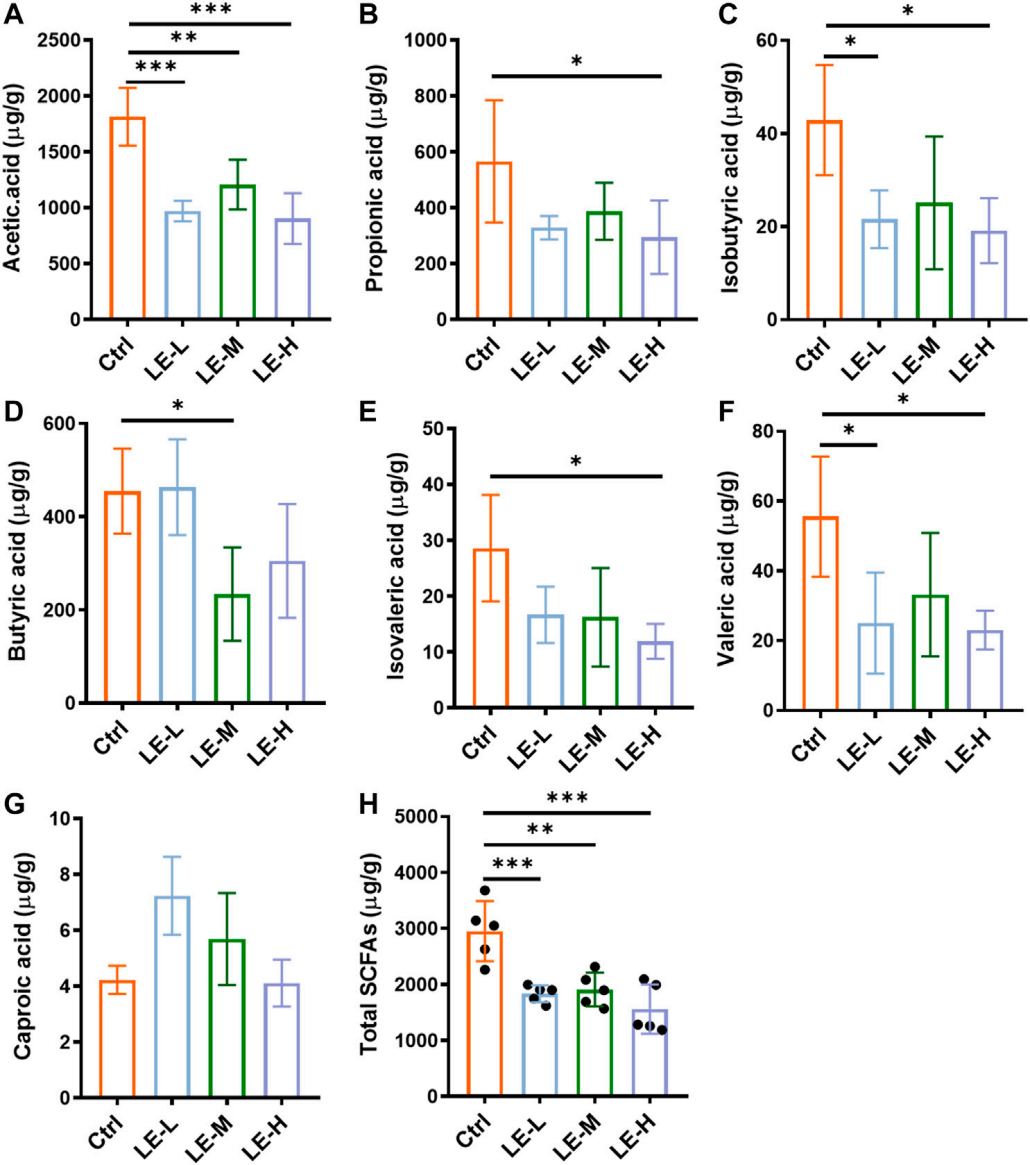
Excessive longan intake alters SCFAs production in mice. Contents of acetic acid **(A)**, propionic acid **(B)**, isobutyric acid **(C)**, butyric acid **(D)**, isovaleric acid **(E)**, valeric acid **(F)**, and caproic acid **(G)** in colonic contents. **(H)** Total contents of SCFAs. Data are presented as mean ± SD (*n* = 5). *p < 0.05, **p < 0.01, ***p < 0.001, compared to Ctrl.

The SCFAs are the end products of bacterial fermentation in gut and have been recognized as mediators of host health (Chambers et al., 2018). We further determined the SCFAs (Acetic acid, propionic acid, isobutyric acid, butyric acid, isovaleric acid, valeric acid and caproic acid) in colonic contents (Figure 3). After a 2 weeks administration of LE, the levels of acetic acid, propionic acid, butyric acid, isobutyric acid, isovaleric acid and valeric acid were significantly reduced in LE-treated group (Figures 3A–F), while caproic acid was not changed in all groups (Figure 3G). The total production of SCFAs in all groups was significantly decreased (Figure 3H) with the lowest level in LE-H group, suggesting the gut homeostasis was influenced.

The results indicated that repeated LE treatments may change gut homeostasis via affecting intestinal microbial communities and related metabolism.

### Excessive Longan Intake Aggravates DSS-Induced Colonic Injury, gut Permeability and Inflammation

**FIGURE 4:**
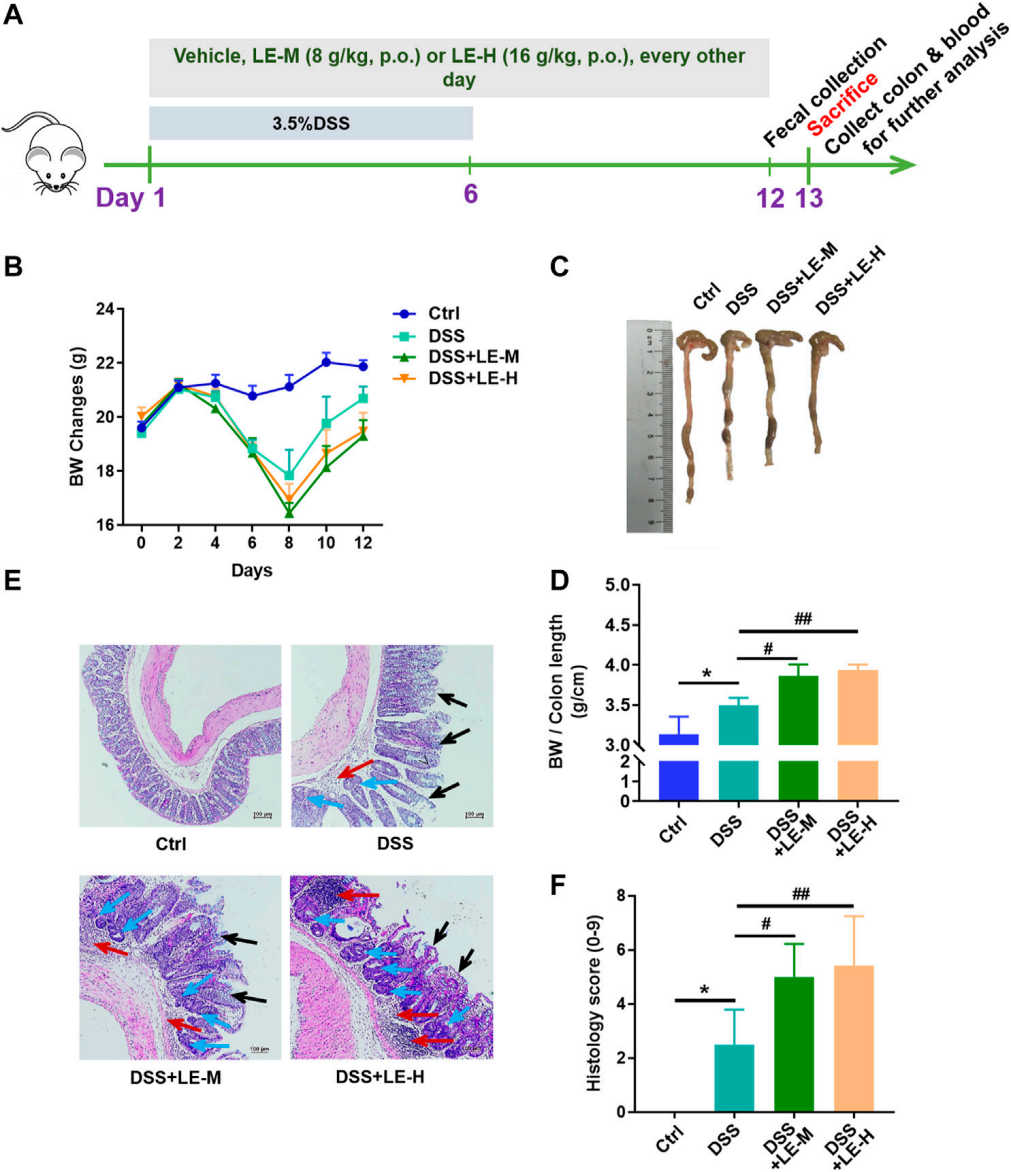
Excessive intake of longan extract (LE) aggravates DSS-induced colitis. **(A)** Experimental design of mouse study **(B)** Body weight changes of mice (*n* = 10) **(C)** Colon length **(D)** Ratio of body weight to colon length (*n* = 7–10) **(E)** Histopathological changes of colon tissues after H and E staining. Red arrow shows inflammatory cell infiltration. Black arrow shows mucous membrane detachment. Blue arrow shows crypt distortion. **(F)** Histological score based on H and E-stained colon sections (*n* = 6). Ctrl, control mice; DSS, 3.5% DSS-treated mice; DSS + LE-M, 3.5% DSS-treated mice supplemented with medium-dose LE (8 g/kg); DSS + LE-H, 3.5% DSS-treated mice supplemented with high-dose LE (16 g/kg). Data are presented as mean ± SD. *p < 0.05, **p < 0.01, ***p < 0.001, compared to Ctrl; ^#^ *p* < 0.05, ^##^ *p* < 0.01, compared to DSS group.

To investigate whether excessive longan intake could coordinate with other pathogenic factors, we established a mouse model of DSS-induced colitis (Figure 4A), and examined the impact of excessive LE intake on this model.

After DSS treatment, mice exhibited a significant weight loss, shortened colon length, and colon injury indicated by increased inflammatory infiltration, crypt distortion and mucous membrane detachment (Figures 4B–F). Compared to DSS treatment alone group, the supplementation of normal dose LE (DSS + LE-L) did not significantly influence the DSS-mediated colonic injury (Supplementary Figure S3). On the contrary, the DSS + LE-M and DSS + LE-H groups showed more severe colonic abnormalities in mice, manifested by the retarded recovery of weight loss (Figure 4B), shorter colon length (Figures 4C,D), more serious histological observations (Figures 4E,F).

**FIGURE 5:**
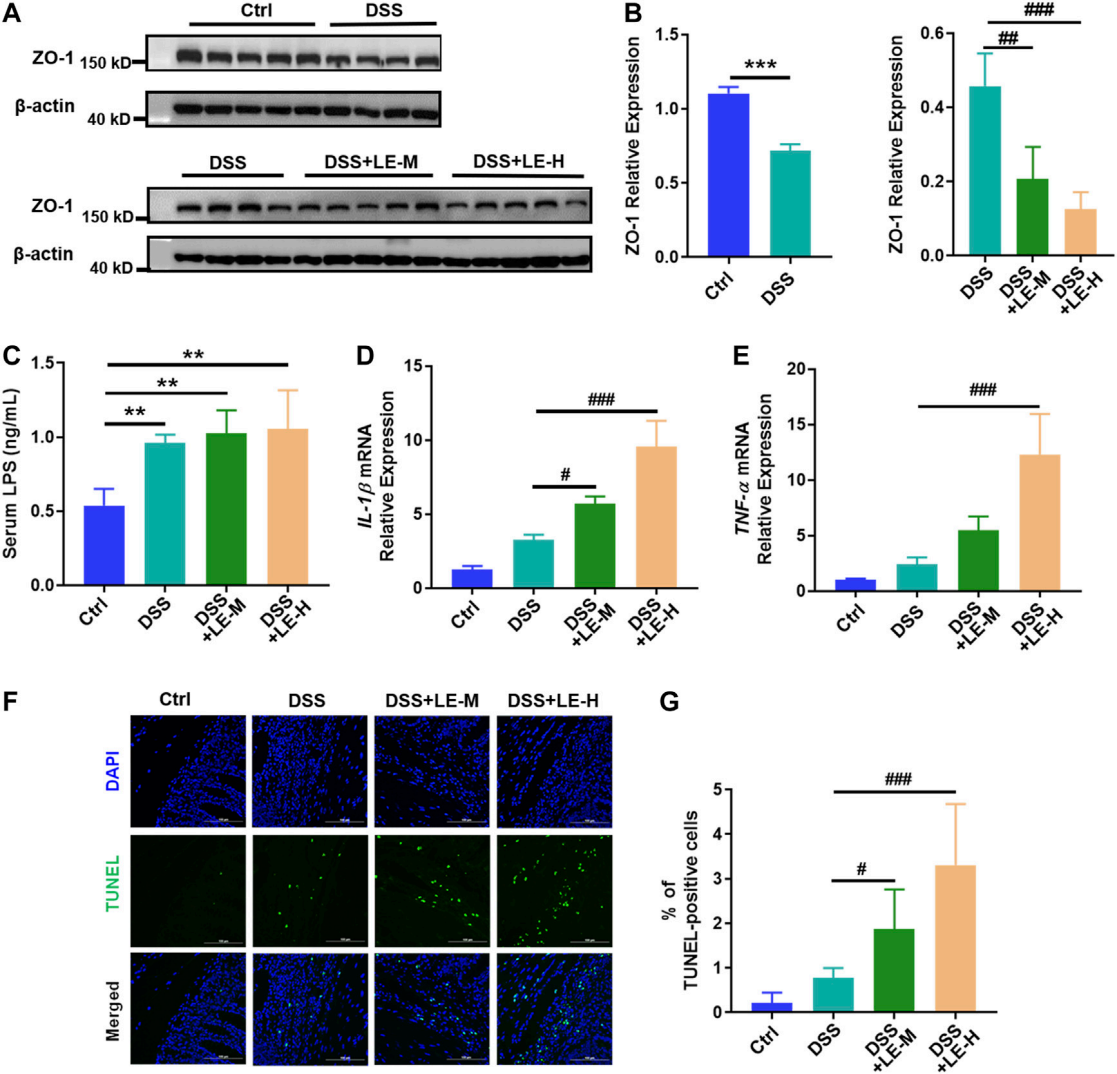
Excessive intake of longan extract (LE) increases gut permeability and enhances colonic injury in colitic mice. **(A)** Protein expression of ZO-1 in colon tissues (*n* = 4–5). Antibody for β-actin was used as internal control. **(B)** Relative expression of ZO-1 over β-actin via gray analysis by ImageJ. **(C)** Serum LPS level (*n* = 4). **(D)** Fold change of *IL-1β* mRNA level in colon tissue (*n* = 4). **(E)** Fold change of *TNF-α* mRNA level in colon tissue (*n* = 4). **(F)** TUNEL staining of colon tissue (all pictures at a × 400 magnification) (*n* = 3). **(G)** Number of TUNEL-positive cells. Ctrl, control mice; DSS, 3.5% DSS-treated mice; DSS + LE-M, 3.5% DSS-treated mice supplemented with medium-dose LE (8 g/kg); DSS + LE-H, 3.5% DSS-treated mice supplemented with high-dose LE (16 g/kg). Data are presented as mean ± SD. *p < 0.05, **p < 0.01, ***p < 0.001, compared to Ctrl; ^#^ *p* < 0.05, ^##^ *p* < 0.01, ^###^ *p* < 0.001, compared to DSS group.

Besides, the protein expression of ZO-1 was reduced in DSS group (Figure 5A, upper panel, and Figure 5B, left panel), and serum level of LPS was elevated (Figure 5C), indicating the increased intestinal permeability in colitic mice. Compared to DSS group, the DSS + LE-M and DSS + LE-H groups revealed a lower expression of ZO-1 (Figure 5A, lower panel, and Figure 5B, right panel), suggesting gut permeability is more serious.

Moreover, DSS induced 2.7 and 2.4-fold of upregulation of gene expression of *IL-1β* and *TNF-α*, respectively, and increased the number of TUNEL positive apoptotic cells in colon by 3.7 times (Figures 5D–G). The combined LE and DSS treatments (DSS + LE-M and DSS + LE-H groups) showed much higher *IL-1β* and *TNF-α* expression (Figures 5D,E) and more TUNEL positive cells (Figure 5F,G), in comparison with DSS group.

Together, the excessive intake of longan, other than the normal dose, exacerbated DSS-induced colonic injury via promoting inflammation and increasing gut permeability in mice.

### Excessive Longan Intake Promotes gut Dysbiosis in DSS-Induced Colitic Mice

**FIGURE 6:**
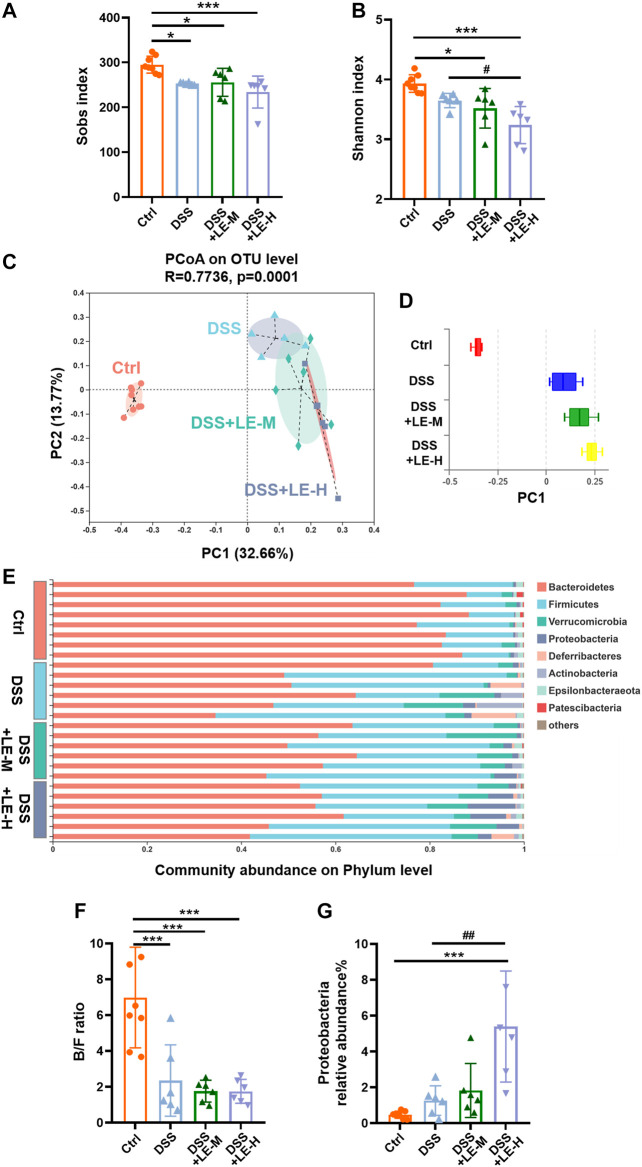
Structural rearrangement of colonic microbial community after excessive longan treatment in colitic mice. **(A)** Sobs index. **(B)** Shannon index. **(C)** PCoA analysis based on OTU level and **(D)** discrete degree of PC1. **(E)** Community abundance on phylum level. **(F)** Ratio of Bacteroidetes/Firmicutes (B/F). **(G)** Relative abundance of Proteobacteria. Data are presented as mean ± SD (*n* = 8 for Ctrl group; *n* = 6 for other groups). *p < 0.05, **p < 0.01, ***p < 0.001, compared to Ctrl; ^#^ *p* < 0.05, ^##^ *p* < 0.01, compared to DSS group.

We further investigated the impact of excessive LE intake on intestinal microbiota in DSS-induced colitic mice. Compared to Ctrl mice, the DSS, DSS + LE-M and DSS + LE-H groups had significantly decreased Sobs index (*p* < 0.05) (Figure 6A), indicating the reduced microbial richness. DSS slightly decreased microbial diversity (reflected by Shannon index), while a significant reduction of Shannon index was further observed in DSS + LE-H group compared to DSS alone group (Figure 6B).

PCoA analysis based on OTU level (Figure 6C) as well as the discrete degree of PC1 (Figure 6D) showed that DSS group was clearly separated from Ctrl group, indicating a structural change of microbial communities. The supplementation of LE in addition to DSS (DSS + LE-M and DSS + LE-H groups) resulted in further alterations in microbial structure. Therefore, the excessive LE may induce specific microbial changes in DSS-mediated colitic mice.

The relative proportions of dominant taxa at the phylum level were determined by microbial taxon assignment in different groups. Bacteroidetes and Firmicutes were the most predominant phyla (Figure 6E). Compared to Ctrl mice, DSS-induced colitic mice had decreased abundance of Bacteroidetes: 83.16% (Ctrl), 54.36% (DSS), 55.67% (DSS + LE-M) and 52.26% (DSS + LE-H), while the Firmicutes level was increased from 13.51% (Ctrl) to 32.67% (DSS), 34.83% (DSS + LE-M), and 33.29% (DSS + LE-H), thus leading to significantly decreased B/F ratio (*p* < 0.001) (Figure 6E,F). Besides, the abundance of Proteobacteria was increased from 0.47% (Ctrl) to 1.26% (DSS), which was further increased to 1.89% (DSS + LE-M) and 5.06% (DSS + LE-H) (Figure 6G). As increase of Proteobacteria has been proposed as a diagnostic marker of dysbiosis and risk of inflammatory bowel disease (IBD) (Shin et al., 2015; Vester-Andersen et al., 2019), the results here indicated that excessive LE treatments promoted gut dysbiosis in DSS-induced colitic mice.

**FIGURE 7:**
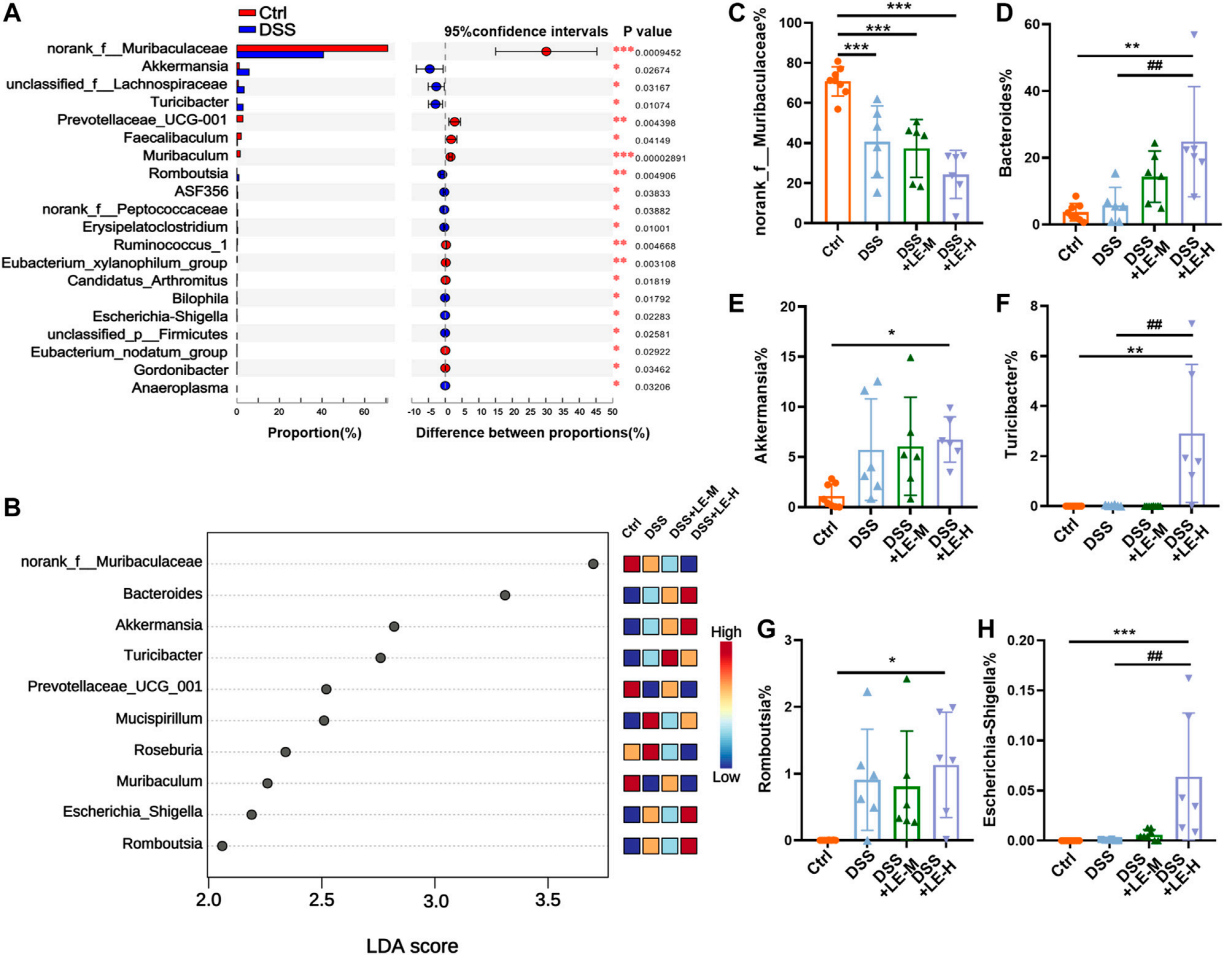
Generic difference among groups after excessive longan treatment in colitic mice. **(A)** Generic difference between Ctrl and DSS group mice. **(B)** Linear discriminant analysis (LDA) score and LDA effect size (LEfSe) analysis (LDA score >2). Generic difference among groups: **(C)** *norank_f__Muribaculaceae* **(D)** *Bacteroides*; **(E)** *Akkermansia*; **(F)** *Turicibacter*; **(G)** *Romboutsia*; **(H)** *Escherichia-Shigella*. Data are presented as mean ± SD (*n* = 8 for Ctrl group; *n* = 6 for other groups). *p < 0.05, **p < 0.01, ***p < 0.001, compared to Ctrl; ^#^ *p* < 0.05, ^##^ *p* < 0.01, compared to DSS group.

At genus level, DSS induced a wide range of microbial alterations (Figure 7A). Nine genera such as the *norank_f__Muribaculaceae*, *Prevotellaceae_UCG-001*, *Faecalibaculum* and *Muribaculum* were significantly decreased, while 11 genera such as *Akkermansia*, *unclassified_f__Lachnospiraceae* and *Turibacter* were significantly increased (Figure 7A). In order to identify the key taxon, LEfSe was performed. A total of 10 genera were identified to be significantly changed among groups with LDA score larger than 2 (Figure 7B).

In particular, *norank_f__Muribaculaceae* was markedly decreased from 70.79% (Ctrl) to 40.63% (DSS), 37.33% (DSS + LE-M), and 24.34% (DSS + LE-H) (Figure 7C). Onthe other hand, the abundance of *Bacteroides* (Figure 7D), *Akkermansia* (Figure 7E), *Turicibacter* (Figure 7F), *Romboutsia* (Figure 7G) and *Escherichia-Shigella* (Figure 7H) were remarkably increased in colitic mice, with the most dramatic elevation observed in DSS + LE-M and/or DSS + LE-H group.

**FIGURE 8:**
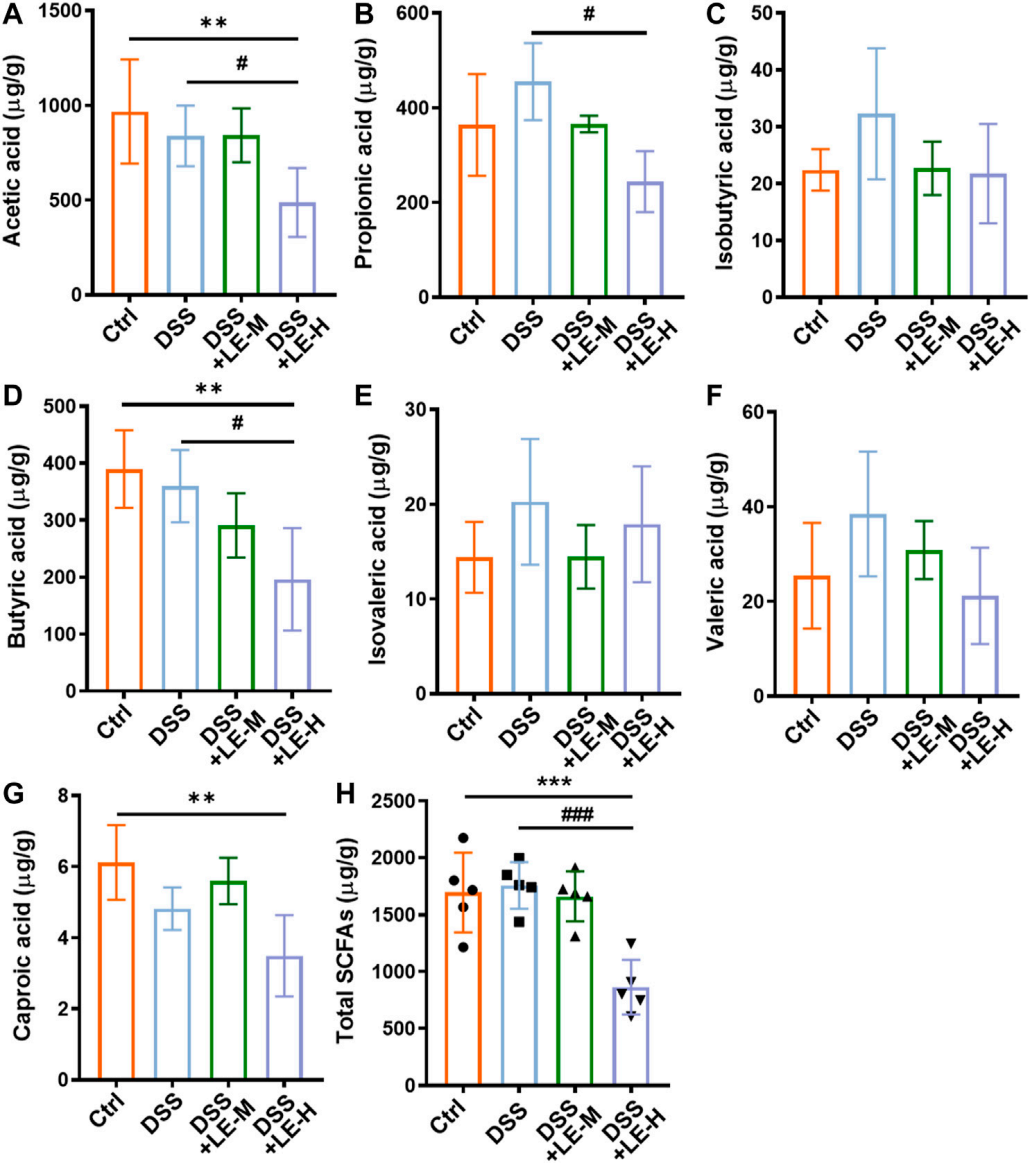
Alteration of SCFA production in colitic mice supplemented with excessive longan. Contents of acetic acid **(A)**, propionic acid **(B)**, isobutyric acid **(C)**, butyric acid **(D)**, isovaleric acid **(E)**, valeric acid **(F)**, and caproic acid **(G)** in colonic contents. **(H)** Total contents of SCFAs. Data are presented as mean ± SD (*n* = 5). *p < 0.05, **p < 0.01, ***p < 0.001, compared to Ctrl. ^#^ *p* < 0.05, ^##^ *p* < 0.01, ^###^ *p* < 0.001, compared to DSS group.

As a result of microbial changes, the contents of SCFAs in LE-treated groups were significantly altered, compared to Ctrl or DSS group (Figure 8). Compared to DSS group, the contents of acetic acid, propionic acid, and butyric acid were significantly decreased in DSS + LE-H group (Figure 8A,B,D), with total SCFAs decreased as well (Figure 8H).

The results indicated that excessive LE treatments (LE-H group in particular) promoted gut dysbiosis and reduced SCFAs production in DSS-induced colitic mice.

### Correlation Analysis of Association of key Microbial Changes and Pathological Abnormities Cross Groups

**FIGURE 9:**
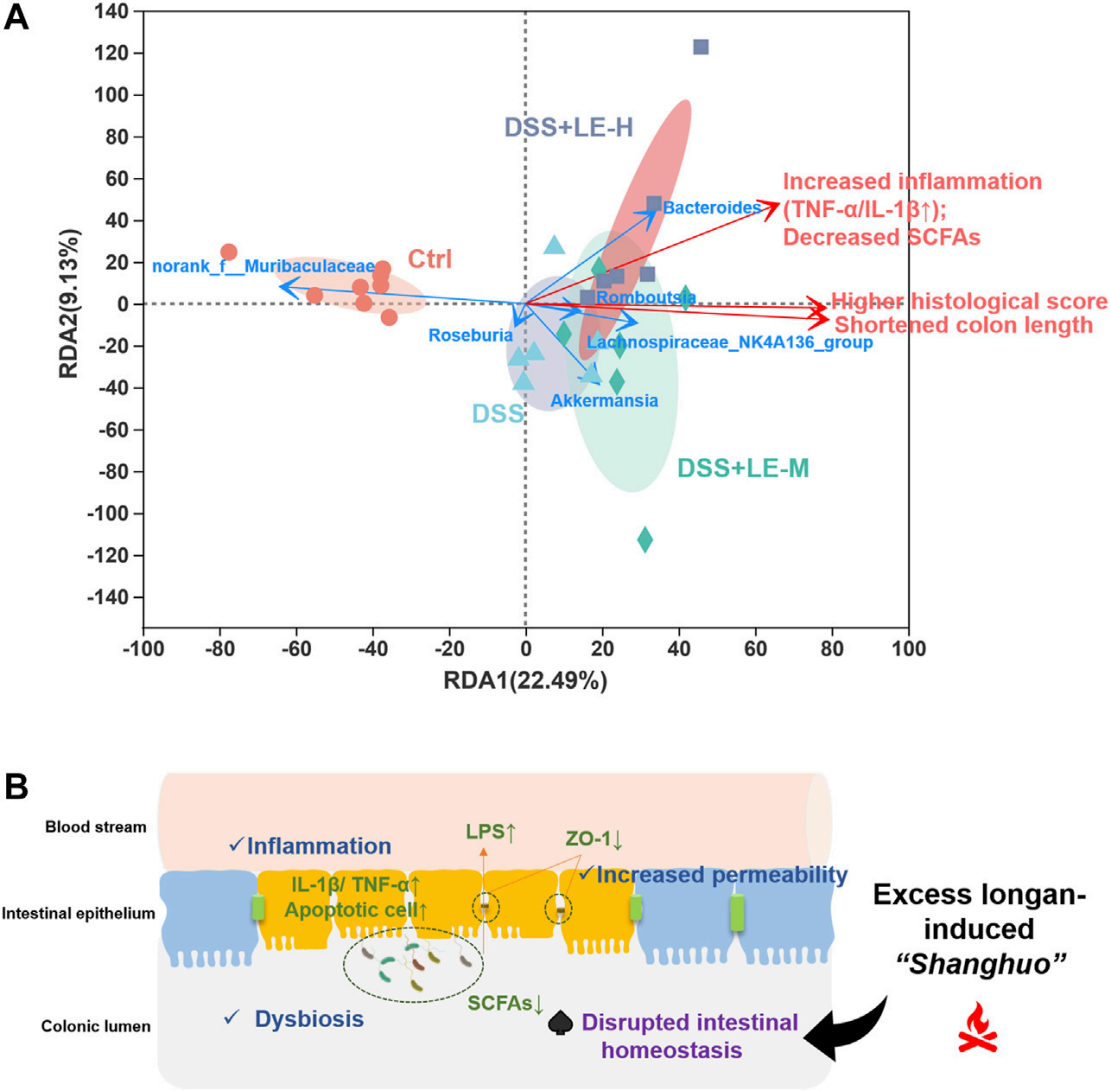
**(A)** Redundancy analysis (RDA) analysis on association of key generic changes and pathological abnormities cross groups **(B)** Schematic illustration of the aggravation of colitis by excessive longan intake and its association with disrupted intestinal homeostasis.

Furthermore, RDA was conducted to summarize the relationships between response variables that can be explained by a set of explanatory variables. As shown in Figure 9A, the direction of colitic mice, especially the mice in DSS + LE-M and DSS + LE-H groups, showed a tendency towards increased histopathological score, elevated inflammation, decreased SCFAs and reduced colon length, and the trend was positively correlated with enriched *Bacteroides*, *Akkermansia*, *Lachnospiraceae_NK4A136_group* and *Romboutsia*, and negatively correlated with *norank_f__Muribaculaceae*.

Collectively, the above results demonstrated that excessive longan intake disrupted intestinal microbiota homeostasis, which could be the underlying mechanism for the aggravated DSS-induced colitis after LE treatment.

## Discussion

Excessive longan intake often causes oral dryness, oral ulcers, gum bleeding and swelling, a status called “*shanghuo*” in traditional Chinese medicine (TCM) system. “*Shanghuo*” related to excessive longan intake is a common phenomenon in daily life. Pan *et al.* highlighted “*shanghuo*” as a promotor for diseases susceptibility (Pan et al., 2020). The aim of this study is to investigate the potential association of excessive intake of longan with the progression of colitis based on the gut homeostasis. Based on the results, we demonstrated for the first time that excessive intake of longan (at 8 and 16 g/kg) significantly exaggerated colitis in mice as evidenced by colonic inflammation, gut permeability as well as histological observations.

We then evaluated the underlying mechanisms. Firstly, excessive longan intake aggravates colitis via disrupting intestinal homeostasis in mice. Disruption in gut homeostasis at several interconnected levels, including the gut microbiome, the microbial metabolites such as SCFAs and endotoxins, and mucus and epithelial barriers, has a profound impact on the pathogenesis of IBD (Albillos et al., 2020). It has long been acknowledged that patients or animals with colitis had dysbiosis with significantly altered gut microbial communities at the phylum, genus and species levels. Dysbiosis led to increased gut permeability, microbial translocation and absorption of microbial products, which increased inflammation and cell injury and altered metabolism (Albillos et al., 2020). In the present study, we firstly demonstrated that excess LE (8 or 16 g/kg) but not the low-dose LE (4 g/kg) supplemented for 2 weeks elevated systemic inflammation in normal mice, observed with structurally changed intestinal microbiome. The altered gut microbiota was characterized by decreased B/F ratio and changed specific microbial communities mainly including the decrease of the nonpathogenic *norank_f__Muribaculaceae* and *Bifidobacterium*, and the increase of *Desulfovibrio* and several genera in *Lachnospiraceae* family such as *unclassified_f__Lachnospiraceae*, *Lachnospiraceae_NK4A136_group* and *norank_f__Lachnospiraceae*, among others. The *norank_f__Muribaculaceae* and *Bifidobacterium* were reportedly potentially beneficial for relieving inflammation, inhibiting harmful bacteria and/or facilitating anticancer immunity (Setoyama et al., 2003; Tang and Yao, 2018; Lv et al., 2019). *Desulfovibrio* can produce the potentially toxic substance of hydrogen sulfide, contributing to gut inflammation which is associated with the pathogenesis of IBD (Carbonero et al., 2012; Mukhopadhya et al., 2012). *Lachnospiraceae* bacteria (especially the *Blautia* and *Roseburia* genus) are generally nonpathogenic and are suggested to produce SCFAs (Vital and Karch, 2017). However, previous studies have implicated that the high-fructose diet resulted in increased abundance of *Desulfovibrio* and the *Lachnospiraceae* family in mice (Do et al., 2018; Li et al., 2019), which was in line with current findings by excess LE treatment containing high sugar contents. The question that how LE-mediated increase of *Lachnospiraceae* family (including *unclassified_f__Lachnospiraceae*, *Lachnospiraceae_NK4A136_group* and *norank_f__Lachnospiraceae*) impacts on SCFA production warrants further investigation. Additionally, previous reports have indicated that SCFAs such as butyric acid and acetic acid were able to inhibit inflammation (Saresella et al., 2020). The results in the present study showed that LE treatment (particularly for the LE-H) reduced acetic acid, butyric acid and isobutyric acid, which may contribute to increased inflammation in mice. Therefore, our results clearly indicated that the excess LE supplementation for 2 weeks could induce inflammation and dysbiosis in mice, which may primarily contribute to “*Shanghuo*”.

Further evidence for the disturbance of intestinal homeostasis by excess LE was obtained on colitic mice. It is demonstrated that excess LE (8 or 16 g/kg) aggravated DSS-induced colitis in mice, showing aggravated inflammation (shorter colon length, upregulated *IL-1β* and *TNF-α*), more serious histological abnormalities, increased gut permeability (decreased ZO-1 protein expression), and increased epithelia injury (increased TUNEL-positive cells) when compared to DSS alone. Moreover, excess LE induced a significant reduction of microbial diversity in colitic mice, accompanied with aggravated alterations of DSS-associated bacteria (Schwab and Berry, 2014; Hakansson et al., 2015; Wang et al., 2019) including the increase of Proteobacteria phylum and genera of *Bacteroides*, *Akkermansia*, *Turicibacter* and *Escherchia-Shigella*, and the decrease of *norank_f__Muribaculaceae*. The changed microbial compositions were accompanied with decreased SCFAs when LE was supplemented with DSS. The increase of Proteobacteria, which contains a variety of pathogens such as *Helicobacter*, *Vibrio* and *Escherchia*, has been proposed as a diagnostic marker of dysbiosis and risk of diseases such as inflammatory bowel disease (Shin et al., 2015; Vester-Andersen et al., 2019). The excess LE-mediated aggravated dysbiosis in colitic mice can be speculated to promote intestinal injury.

Furthermore, due to a high content of free sugars (fructose, 17.6%; glucose, 13.7%; and sucrose, 37.2%) in longan, longan supplementation in the present study was accompanied with excessive free sugar intake. Sucrose, a disaccharide, is hydrolyzed in gut into glucose and fructose prior to absorption. Excess dietary free sugars have been demonstrated to promote colitis in mice via altering gut microbiota (Khan et al., 2020). In this study, the excess doses for longan used in mice are 8 g/kg (40–56 g human equivalents) and 16 g/kg (80–112 g human equivalents), which have exceeded the WHO-recommended daily intake of free sugars (less than 25–50 g; equal to 36–72 g Longan) for children or adults (WHO, 2015). It is speculated that excess free sugars, accompanied with LE supplementation, have led to impaired colonic homeostasis. Therefore, the high level of free sugars in longan may contribute to the aggravation of colitis in mice.

## Conclusion

In this study, we provided the first evidence that the excessive longan supplementation (8 and 16 g/kg) significantly aggravated colitis in mice, which was tightly associated with the disruption of intestinal homeostasis (Figure 9B). The results provided a scientific basis for excessive longan-induced abnormal body status (“*Shanghuo*”) via disrupting gut homeostasis. Our findings warrant rational longan (especially for the dried longan) consumption as a dietary supplement among the general population and suggest contraindications such as IBD of using longan as an herbal medicine.

## Data Availability

The datasets presented in this study can be found in online repositories. The names of the repository/repositories and accession number(s) can be found below: NCBI BioProject, accession no: PRJNA699680.

## Abbreviations

DSS, dextran sulfate sodium; ELSD, evaporative light scattering detector; HFD, high-fat diet; IBD, inflammatory bowel disease; Longan, Arillus Longan; LE, Longan extract; LDA, linear discriminant analysis; LEfSe, linear discriminant analysis coupled with effect size; NAFLD, non-alcoholic fatty liver disease; OTU, operational taxonomic unit; PCoA, principal co-ordinates analysis; PLS-DA, partial least squares discriminant analysis; SCFA, short-chain fatty acid; TCM, traditional Chinese medicine; WHO, World Health Organization.

## References

[B1] Albillos A. de Gottardi A. Rescigno M. (2020). The gut-liver axis in liver disease: pathophysiological basis for therapy. J. Hepatol. 72, 558–577. 10.1016/j.jhep.2019.10.003 31622696

[B4] Bray G. A. Popkin B. M. (2014). Dietary sugar and body weight: have we reached a crisis in the epidemic of obesity and diabetes? Diabetes Care 37, 950–956. 10.2337/dc13-2085 24652725 PMC9514031

[B5] Carbonero F. Benefiel A. C. Alizadeh-Ghamsari A. H. Gaskins H. R. (2012). Microbial pathways in colonic sulfur metabolism and links with health and disease. Front Physiol. 3, 448. 10.3389/fphys.2012.00448 23226130 PMC3508456

[B6] Chambers E. S. Preston T. Frost G. Morrison D. J. (2018). Role of gut microbiota-generated short-chain fatty acids in metabolic and cardiovascular health. Curr. Nutr. Rep. 7, 198–206. 10.1007/s13668-018-0248-8 30264354 PMC6244749

[B7] Chazelas E. Srour B. Desmetz E. Kesse-Guyot E. Julia C. Deschamps V. (2019). Sugary drink consumption and risk of cancer: results from nutrinet-santé prospective cohort. Br. Med. J. 366, l2408. 10.1136/bmj.l2408 31292122 PMC6614796

[B8] Chen J. Sun X.-D. Wang Y. Zhou L.-M. (2010). Effect of polysaccharides of the *Euphoria longan* (Lour.) Steud on inflammatory response induced by focal cerebral ischemia/reperfusion injury in rats. Food Agr. Immunol. 21, 219–225. 10.3109/02699052.2010.546824

[B9] Cheng H. Huo J. (2009). One case of allergy due to overintake of logan. People's Mil. Surg. 52, 621.

[B10] Chiranthanut N. Teekachunhatean S. Panthong A. Lertprasertsuke N. (2020). Acute and chronic oral toxicity assessment of longan sugar extracts derived from whole fruit and from fruit pulp in rats. J. Ethnopharmacol. 263, 113184. 10.1016/j.jep.2020.113184 32736055

[B13] Do M. H. Lee E. Oh M. J. Kim Y. Park H.-Y. (2018). High-glucose or fructose diet cause changes of the gut microbiota and metabolic disorders in mice without body weight change. Nutrients 10, 761. 10.3390/nu10060761 PMC6024874 29899272

[B15] Hakansson A. Tormo-Badia N. Baridi A. Xu J. Molin G. Hagslätt M. L. (2015). Immunological alteration and changes of gut microbiota after dextran sulfate sodium (DSS) administration in mice. Clin. Exp. Med. 15, 107–120. 10.1007/s10238-013-0270-5 24414342 PMC4308640

[B17] Khan S. Waliullah S. Godfrey V. Khan M. A. W. Ramachandran R. A. Cantarel B. L. (2020). Dietary simple sugars alter microbial ecology in the gut and promote colitis in mice. Sci. Transl. Med. 12, eaay6218. 10.1126/scitranslmed.aay6218 33115951

[B20] Li J. M. Yu R. Zhang L. P. Wen S. Y. Wang S. J. Zhang X. Y. (2019). Dietary fructose-induced gut dysbiosis promotes mouse hippocampal neuroinflammation: a benefit of short-chain fatty acids. Microbiome 7, 98. 10.1186/s40168-019-0713-7 31255176 PMC6599330

[B22] Li Y. (2012). Determination of polysaccharide content in longan from different areas. J. Gansu Univ. Chin. Med. 29, 59–60.

[B26] Lv J. Jia Y. Li J. Kuai W. Li Y. Guo F. (2019). Gegen qinlian decoction enhances the effect of PD-1 blockade in colorectal cancer with microsatellite stability by remodelling the gut microbiota and the tumour microenvironment. Cell Death Dis. 10, 415. 10.1038/s41419-019-1638-6 31138779 PMC6538740

[B27] Mukhopadhya I. Hansen R. El-Omar E. M. Hold G. L. (2012). IBD—what role do Proteobacteria play? Nat. Rev. Gastroenterol. Hepatol. 9, 219–230. 10.1038/nrgastro.2012.14 22349170

[B28] Pan M.-H. Zhu S. R. Duan W. J. Ma X. H. Luo X. Liu B. (2020). “Shanghuo” increases disease susceptibility: modern significance of an old TCM theory. J. Ethnopharmacol 250, 112491. 10.1016/j.jep.2019.112491 31863858

[B29] Park S. J. Park D. H. Kim D. H. Lee S. Yoon B. H. Jung W. Y. (2010). The memory-enhancing effects of Euphoria longan fruit extract in mice. J. Ethnopharmacol. 128, 160–165. 10.1016/j.jep.2019.112491 20064595

[B30] Rongrong H. Hiroshi K. (2008). Shanghuo syndrome in traditional Chinese medicine. World Sci. Technol. 10, 37–41. 10.1016/S1876-3553(09)60024-7

[B31] Saresella M. Marventano I. Barone M. La Rosa F. Piancone F. Mendozzi L. (2020). Alterations in circulating fatty acid are associated with gut microbiota dysbiosis and inflammation in multiple sclerosis. Front Immunol. 11, 1390. 10.3389/fimmu.2020.01390 32733460 PMC7358580

[B32] Schwab C. Berry D. (2014). Longitudinal study of murine microbiota activity and interactions with the host during acute inflammation and recovery. ISME J. 8, 1101–1114. 10.1038/ismej.2013.223 24401855 PMC3996699

[B33] Setoyama H. Imaoka A. Ishikawa H. Umesaki Y. (2003). Prevention of gut inflammation by Bifidobacterium in dextran sulfate-treated gnotobiotic mice associated with Bacteroides strains isolated from ulcerative colitis patients. Microbes Infect. 5, 115–122. 10.1016/s1286-4579(02)00080-1 12650769

[B34] Shin N.-R. Whon T. W. Bae J. W. (2015). Proteobacteria: microbial signature of dysbiosis in gut microbiota. Trend Biotechnol. 33, 496–503. 10.1016/j.tibtech.2015.06.011 26210164

[B35] Tang W. Yao X. (2018). Modulation of the gut microbiota in rats by Hugan Qingzhi tablets during the treatment of high-fat-diet-induced nonalcoholic fatty liver disease. Oxid. Med. Cell Longev. 2018, 7261619. 10.1155/2018/7261619 30671174 PMC6323444

[B36] Te Morenga L. Mallard S. Mann J. (2013). Dietary sugars and body weight: systematic review and meta-analyses of randomised controlled trials and cohort studies. Br. Med. J. 346, e7492. 10.1136/bmj.e7492 23321486

[B39] Todoric J. Di Caro G. Reibe S. Henstridge D. C. Green C. R. Vrbanac A. (2020). Fructose stimulated de novo lipogenesis is promoted by inflammation. Nat. Metab. 2, 1034–1045. 10.1038/s42255-020-0261-2 32839596 PMC8018782

[B40] Turnbaugh P. J. Hamady M. Yatsunenko T. Cantarel B. L. Duncan A. Ley R. E. (2009). A core gut microbiome in obese and lean twins. Nature 457, 480–484. 10.1038/nature07540 19043404 PMC2677729

[B41] Vester-Andersen M. K. Mirsepasi-Lauridsen H. C. Prosberg M. V. Mortensen C. O. Träger C. Skovsen K. (2019). Increased abundance of *Proteobacteria* in aggressive crohn’s disease seven years after diagnosis. Sci. Rep. 9, 13473. 10.1038/s41598-019-49833-3 31530835 PMC6748953

[B42] Vital M. Karch A. (2017). Colonic butyrate-producing communities in humans: an overview using omics data. mSystems 2, e00130–17. 10.1128/mSystems.00130-17 29238752 PMC5715108

[B43] Wang S. L. Shao B. Z. Zhao S.-B. Chang X. Wang P. Miao C. Y. (2019). Intestinal autophagy links psychosocial stress with gut microbiota to promote inflammatory bowel disease. Cell Death Dis. 10, 391. 10.1038/s41419-019-1634-x 31564717 PMC6766473

[B44] WHO (2015). Guideline: sugars intake for adult and children. Geneva, Switzerland: World Health Organization, 49. 25905159

[B45] Yin J. Ren W. Wei B. Huang H. Li M. Wu X. (2020). Characterization of chemical composition and prebiotic effect of a dietary medicinal plant *Penthorum chinense* Pursh. Food Chem. 319, 126568. 10.1016/j.foodchem.2020.126568 32169768

[B46] Zhang R. Khan S. A. Lin Y. Guo D. Pan X. Liu L. (2018). Phenolic profiles and cellular antioxidant activity of longan pulp of representative Chinese cultivars. Int. J. Food Prop. 21, 746–759. 10.1080/10942912.2018.1425705

[B47] Zhang X. Guo S. Ho C.-T. Bai N. (2020). Phytochemical constituents and biological activities of longan (*Dimocarpus longan Lour*) fruit: a review. Food Sci. Hum. Well 9, 95–102. 10.1016/j.fshw.2020.03.001

[B48] Zhao S. Jang C. Liu J. Uehara K. Gilbert M. Izzo L. (2020). Dietary fructose feeds hepatic lipogenesis via microbiota-derived acetate. Nature 579, 586–591. 10.1038/s41586-020-2101-7 32214246 PMC7416516

[B49] Zhong M. Rao W. Xiao C. (2011). HPLC-ELSD method for quantification of fructose, glucose and sucrose in longan. Drug Stand. China 12, 44–48. 10.19778/j.chp.2011.01.015

[B50] Zhu L. Xue J. Xia Q. Fu P. P. Lin G. (2017). The long persistence of pyrrolizidine alkaloid-derived DNA adducts *in vivo*: kinetic study following single and multiple exposures in male ICR mice. Arch. Toxicol. 91, 949. 10.1007/s00204-016-1713-z 27125825

