# Supplementary material for: Excessive Intake of Longan Arillus Alters gut Homeostasis and Aggravates Colitis in Mice
Source: Front Pharmacol. 2021 Mar 26;12:640417. doi: 10.3389/fphar.2021.640417 (PMC8033040; doi:10.3389/fphar.2021.640417)
Supplement: Supplementary file 1 [file datasheet1.docx]

**Table S1.** The results of linearity, limits of quantification (LOQs), precision and repeatability for detection of each analyte.

| Analyte | Rt. (min) | Detection ion (*m/z*) | Linearity | | | LOQs (μg/mL) | Intra-day precision (RSD, %) | Inter-day precision (RSD, %) | Repeatability (RSD, %) |
| --- | --- | --- | --- | --- | --- | --- | --- | --- | --- |
|  |  |  | Calibration curves | Coeffient (r) | Range (μg/mL) |  |  |  |  |
| acetic acid | 4.99 | 60 | y=0.039x+0.0025 | 0.9919 | 0.02-500 | 0.02 | 1.85 | 11.73 | 5.44 |
| propionic acid | 6.08 | 74 | y=0.0351x+0.0014 | .09937 | 0.02-500 | 0.02 | 1.73 | 5.28 | 4.72 |
| isobutyric acid | 6.47 | 73 | y=0.0492x+0.001 | 0.9939 | 0.02-500 | 0.02 | 1.31 | 4.76 | 5.65 |
| butyric acid | 7.4 | 60 | y=0.1052x+0.0011 | 0.9964 | 0.02-500 | 0.02 | 1.57 | 6.87 | 5.51 |
| isovaleric acid | 8.06 | 60 | y=0.1302x+7e-04 | 0.9969 | 0.02-500 | 0.02 | 1.35 | 6.63 | 7.59 |
| valeric acid | 9.22 | 60 | y=0.1142x+2e-04 | 0.9966 | 0.02-500 | 0.02 | 1.30 | 5.66 | 7.68 |
| hexanic acid | 10.49 | 60 | y=0.0972x+0.0017 | 0.9964 | 0.02-500 | 0.02 | 1.00 | 7.71 | 9.53 |

Notes: x represents concentration while y represents the peak area ratio of each analyte over that of the internal standard. Intraday precision was performed by consecutively analyzing the quality control (QC) samples at 25 μg/mL for 8 times. Interday precision was conducted by analyzing the QC samples at 25 μg/mL for three consecutive days. Repeatability was conducted by analyzing 6 independently-processed samples of same origin. Rt, retention time.

**Table S2.** The results of recovery for detection of each analyte.

| Analyte |  | Recovery (%) | | |
| --- | --- | --- | --- | --- |
|  |  | QC1 | QC2 | QC3 |
| acetic acid |  | 90.34 | 97.25 | 106.31 |
| propionic acid |  | 94.55 | 87.81 | 87.53 |
| isobutyric acid |  | 107.07 | 86.17 | 85.20 |
| butyric acid |  | 96.35 | 85.56 | 86.38 |
| isovaleric acid |  | 92.82 | 85.50 | 85.80 |
| valeric acid |  | 99.25 | 88.16 | 89.19 |
| hexanic acid |  | 85.22 | 96.97 | 96.76 |

Notes: QC1, 1 μg/mL; QC2, 25 μg/mL; QC3, 100 μg/mL. Recovery was conducted by analyzing different QC samples by developed method and calculated by: Recovery (%) = (detected quantity - actual quantity) / actual quantity * 100%.


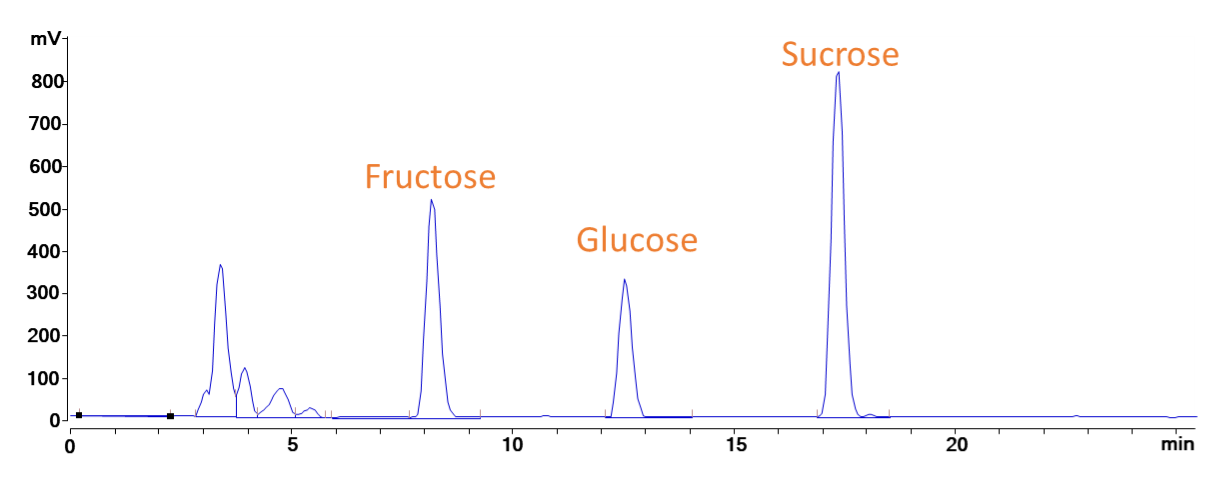


**Fig. S1.** HPLC chromatogram for longan extract. The quantification of fructose, glucose and sucrose in longan was performed on an Agilent liquid chromatograph system (Agilent Technologies, USA) coupled with an Alltech 2000 evaporative light scattering detector (ELSD; Grace, USA). Chromatographic separation was achieved on an Asahipak NH2P-504E column (4.6 mm × 250 mm, Japan). The mobile phases consisted of water and acetonitrile (v/v, 25:75) at a flow rate of 1 mL/min. The drift tube temperature was set at 110 °C, with nitrogen flow rate at 2.5 L/min. The injection volume was 10 μL.


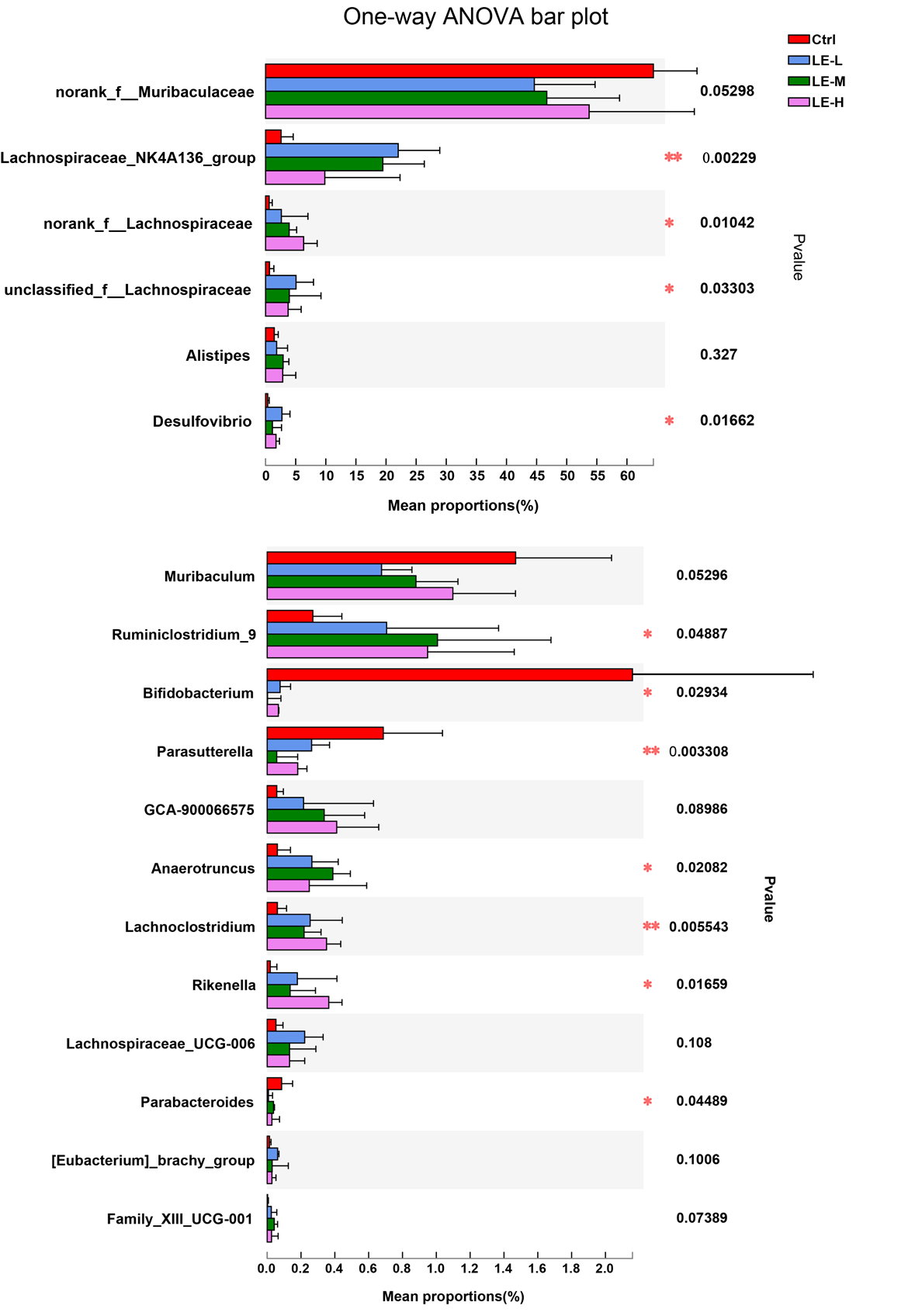


**Fig. S2.** Generic difference among groups after 2 weeks of *Longan* extract treatment in normal mice. **p* < 0.05, ***p* < 0.01, by one-way ANOVA.


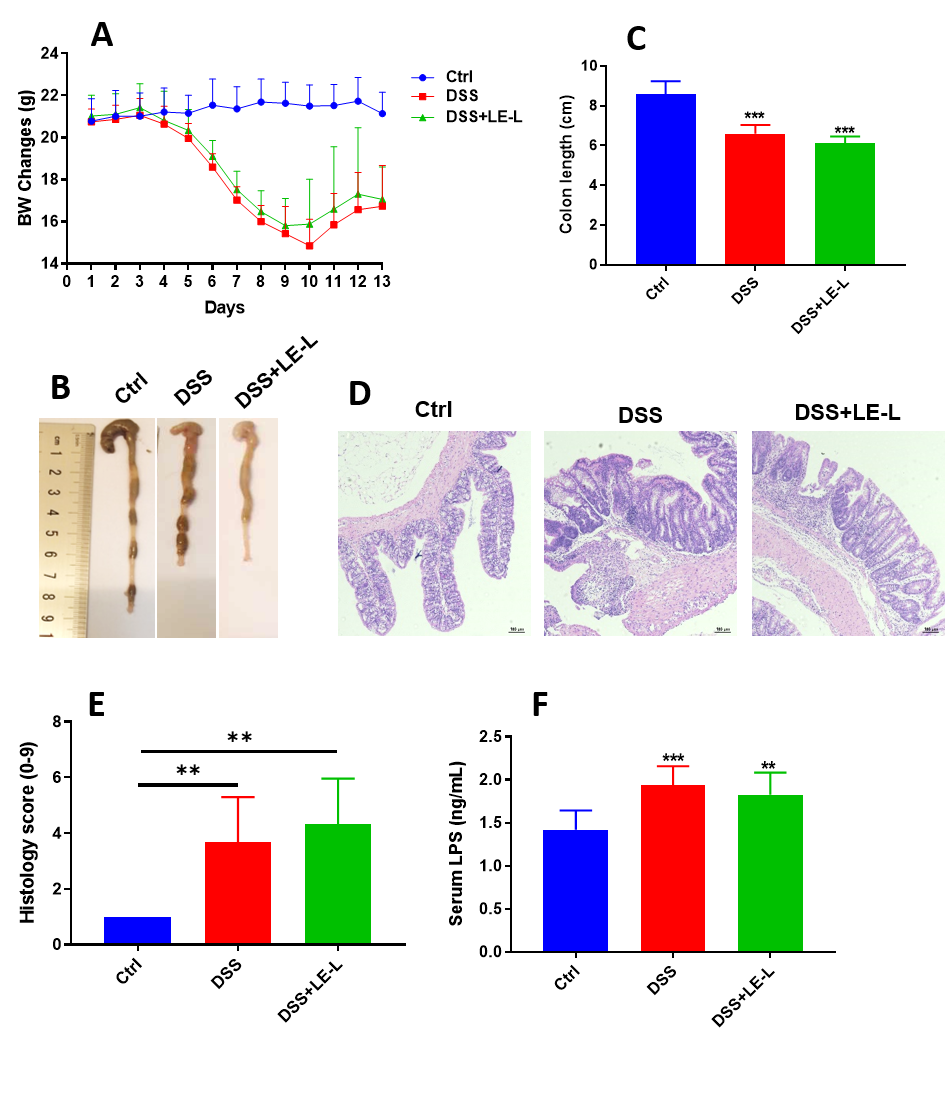
 **Fig. S3.** Intake of longan extract (LE) at 4 g/kg did not aggravate DSS-induced colitis in mice. (A) Body weight changes. (B) Colon length. (C) Statistics for colon length. (D) Histopathological changes of colon tissues after H&E staining. (E) Histological score based on H&E stained colon sections. (F) Serum level of LPS. Data are presented as mean ± SD. **p* < 0.05, ***p* < 0.01, compared to Ctrl.
